# Exploring the Antecedents and Outcomes of Entrepreneurial Well-Being: Empirical Evidence From Iranian Rural Entrepreneurs

**DOI:** 10.3389/fpsyg.2022.858230

**Published:** 2022-03-14

**Authors:** Saeid Karimi, Sima Reisi

**Affiliations:** Department of Agricultural Extension and Education, Faculty of Agriculture, Bu-Ali Sina University, Hamedan, Iran

**Keywords:** job fit, entrepreneurial passion, well-being, entrepreneurial persistence, rural entrepreneurship

## Abstract

Studies regarding rural entrepreneurs in developing countries and, specifically, their well-being have not been sufficiently developed in the literature. The present study was an attempt to fill this gap and to examine important antecedents and outcomes associated with entrepreneurial well-being among a sample of 102 Iranian rural entrepreneurs. This research established a theoretical research model that highlighted the relationships among job fit, entrepreneurial passion, well-being, and persistence. The results of structural equation modeling supported the hypothesized model. In particular, the results indicated that higher job fit was associated with higher levels of entrepreneurial well-being through an entrepreneurial passion mechanism. Moreover, the results supported the hypothesized serial mediating effect. In other words, a high level of job fit enhances entrepreneurial persistence through a high level of entrepreneurial passion and well-being. This study provides significant input to policymakers and practitioners in their efforts to promote the antecedents and outcomes of well-being among rural entrepreneurs.

## Introduction

Growing attention has been paid to entrepreneurs’ well-being in recent years as it is linked with various positive outcomes such as more efficiency, better performance, and increased opportunity recognition (Stephan, 2018; Wiklund et al., 2019; Marshall et al., 2020). Furthermore, entrepreneurs view their well-being as an index of their success (Wach et al., 2016). Entrepreneurial well-being represents the experience of satisfaction, positive affect, non-frequent negative affect, and a psychological function regarding initiation, growth, and administration of a business. Some researchers argue that entrepreneurial well-being can be more helpful in better understanding entrepreneurs’ behavior and performance (e.g., Shepherd et al., 2019; Marshall et al., 2020). They believe that an entrepreneur’s well-being has important consequences for business and entrepreneurs personally value their well-being. Therefore, researchers have investigated well-being as an entrepreneurial outcome and have focused on factors and mechanisms that can influence entrepreneurs’ mental health in recent years (Stephan et al., 2022). Nonetheless, robust theories have not been well developed for studying the factors underpinning entrepreneurs’ well-being yet and there is not a full understanding of its antecedents and outcomes (Shir et al., 2019; Lanivich et al., 2021). Recent research studies have revealed that various factors influence entrepreneurs’ well-being, such as business type, personality traits, and environmental conditions (e.g., Stephan, 2018; Marshall et al., 2020). While we know that various factors are involved in entrepreneurial well-being, one of the most important factors that can affect people’s well-being is their job fit (Lin et al., 2014; Choi et al., 2017). Job fit can be defined as the extent to which an individual’s current job matches her ideal job (Burnette and Pollack, 2013). Research shows that people who perceive higher fit with their job have less stress and exhaustion, more positive work experiences, greater job satisfaction, and better performance (Kristof-Brown et al., 2005; Peng and Mao, 2015). Interestingly, despite the growing number of studies on entrepreneurial well-being, little attention has been paid to the effect of job fit. So, the present study aims to relate job fit to entrepreneurs’ well-being both conceptually and empirically.

This research uses entrepreneurial passion in the attempt to account for the mechanism between job fit and well-being. Entrepreneurial passion is regarded as the heart of entrepreneurship and can be a vital component in entrepreneurial behavior and the process of business creation and its outcomes (Cardon et al., 2013; Santos and Cardon, 2019; Karimi, 2020). According to Vallerand et al. (2003), passion is accompanied by a strong emotion for doing a job that people would like to attain with sheer energy. Researchers suggest that entrepreneurial passion plays a crucial role in the entrepreneurial process (Karimi, 2020). Surprisingly, no research has ever investigated the relationship between entrepreneurial passion and well-being, although a positive relationship can be expected between them (Cardon et al., 2009; Stephan, 2018).

Additionally, in entrepreneurial well-being studies, there are very few theoretical and empirical models that express how well-being produces entrepreneurial action (Wiklund et al., 2019). Although many researchers argue that well-being may increase an entrepreneur’s determination to persist through the future challenges of the business, few studies have related these two constructs empirically (Marshall et al., 2020). Persistence is a key element in entrepreneurship because launching and developing a venture is an ambitious and arduous process faced with many barriers (Cardon and Kirk, 2015). Research has shown that entrepreneurs who are pertinacious in pursuing their goals are more likely to succeed (Timmons and Spinelli, 2009). So, it is imperative to explore the factors influencing entrepreneurial persistence (Cardon and Kirk, 2015), which is another goal of the present study.

The present research helps extend the literature and has important applications for research and practice in the field of entrepreneurial well-being. Based on the findings of previous studies, the research develops a theoretical model in which job fit affects entrepreneurs’ well-being through entrepreneurial passion. The model also helps resolve a weakness in the research literature. The model emphasizes that well-being is not only an outcome variable (Shepherd et al., 2019) but also a likely mediator variable in entrepreneurial activity models (Marshall et al., 2020). Indeed, the researchers regret that research in which well-being is studied as a source or incentive of entrepreneurial activity is still limited, so new studies may help us understand well-being as a necessary source and psychological mechanism in entrepreneurship (Wiklund et al., 2019, p. 584). In this research, entrepreneurial well-being acts as a mediator variable between entrepreneurial passion and entrepreneurial persistence, so it contributes to improving the research literature on entrepreneurial well-being, passion, and persistence. In general, the research model expands the research on job fit, passion, well-being, and entrepreneurial persistence by explaining the processes that relate perceptions of job fit to the behavioral outcomes of entrepreneurial persistence.

To the best knowledge of the authors, there is little research on rural entrepreneurs’ well-being in developing countries. Most previous studies have been conducted on urban entrepreneurs’ well-being and/or in developed countries (e.g., Gilbert et al., 2016; Marshall et al., 2020; Nikolaev et al., 2020). Recent studies suggest that entrepreneurs’ well-being and factors affecting it vary across rural and urban settings (Pham et al., 2018; Abreu et al., 2019). Thus, exploring the antecedents and outcomes of rural entrepreneurs’ well-being contributes to the entrepreneurial well-being literature, especially in developing countries. Based on the literature (e.g., Sutter et al., 2019; Dong et al., 2021), entrepreneurship can contribute significantly to the poverty alleviation and well-being of rural communities. Nonetheless, there is limited empirical evidence on rural entrepreneurship and especially rural entrepreneurs’ well-being while it can help policymakers and practitioners who aim to reduce poverty and improve welfare in rural areas (Bhuiyan and Ivlevs, 2019). Accordingly, this study attempted to provide insight into rural entrepreneurs’ well-being.

## Theoretical Background and Hypotheses

### Theory of Subjective Well-Being and Entrepreneurship

Subjective well-being, or simply well-being, is a term commonly used in various scientific disciplines. It not only includes attaining joy and avoiding pain (hedonism perspective) but also emphasizes vivacity, meaning, and self-actualization of mental health (eudaimonic perspective) (Wiklund et al., 2019). Given the nature of the term well-being and its consequences and application, it is also used instead of happiness, life satisfaction, and life quality. Many researchers define subjective well-being as “the extent to which people are happy with their life and job” (Deng et al., 2019; Marshall et al., 2020).

It is crucial to understand well-being incentives in different research fields because, as Aristotle claims, it may be the ultimate goal of human existence (Marshall et al., 2020). Researchers have paid growing attention to subjective well-being in entrepreneurship and its underpinning factors in recent years (e.g., Uy et al., 2013; Ryff, 2019; Shir et al., 2019; Wiklund et al., 2019; Nikolaev et al., 2020). Based on the research, various personal and environmental factors such as the work conditions and personal traits of entrepreneurs can influence their well-being (Stephan, 2018; Marshall et al., 2020). For instance, Shir et al. (2019) and Lanivich et al. (2021) used self-determination theory to develop a research model and explore the relationship between the satisfaction of entrepreneurs’ three basic psychological needs – autonomy, competence, and relatedness – and their well-being. Marshall et al. (2020) theorized in their study that feelings of well-being for entrepreneurs with access to resources and self-efficacy will lead to greater persistence with startup activities. Nikolaev et al. (2020) developed and tested a model in which psychological functioning mediated the relationship between entrepreneurship and subjective well-being. de Mol et al. (2018) developed and examined a model of entrepreneurial burnout that highlighted the relationships among job fit, entrepreneurial passion, destiny beliefs, and burnout. Their findings indicated that job fit perceptions were positively related to passion. However, to the best knowledge of the authors, no research has ever investigated the relationship of job fit and entrepreneurial passion with entrepreneurs’ subjective well-being and persistence.

### Job Fit and Entrepreneurial Passion

Entrepreneurial passion is strong positive emotions toward entrepreneurial activities and is important for the entrepreneur’s self-similarity (Cardon et al., 2009). Passion helps entrepreneurs persist in the sophisticated entrepreneurial process. It is also like strong internal determination that makes possible any impossible (Murnieks et al., 2014) and is a strong motivator for hard work, dedication, and intention toward making changes (Cardon et al., 2013). Indeed, entrepreneurial passion is the driving force that guides the process by which entrepreneurial ideas are converted and adapted to real-world conditions to achieve success. The insight gained by this process is continuously reinforced and manages the actualization of business ideas (Chen et al., 2009).

Research has shown that entrepreneurial passion is invaluable in accounting for work-related outcomes (e.g., Ho and Pollack, 2014; Murnieks et al., 2014; Cardon and Kirk, 2015). According to de Mol et al. (2018), job fit affects both cognitive and affective components of entrepreneurial passion for various reasons. First, it has been shown that job-person fit has a positive relationship with job satisfaction (Verquer et al., 2003) so that entrepreneurs who perceive that their entrepreneurial job matches their ideal job are more likely to enjoy and like their jobs (i.e., the affective component of passion). They do the job because their job gives them the feeling of delight and satisfaction when they are engaged with it. Second, in proportion to the extent the entrepreneurial activities they are engaged in match their perception of an ideal job, they may internalize the entrepreneurial activity and identify themselves with it (i.e., the cognitive component of passion) so that people are more likely to relate their personal identity to their entrepreneurial job. So, both affective and identity-oriented reasons suggest that entrepreneurial job fit is positively associated with entrepreneurial passion. This relationship is supported by the results of de Mol et al. (2018), too.

**H1:** Entrepreneurial job fit is positively related to entrepreneurial passion.

### Entrepreneurial Passion and Subjective Well-Being

Researchers suggest that people’s career can influence their well-being and satisfaction with their personal and family life. So, passion for a job or certain activity is expected to influence the person’s life. Passion for a job motivates the person to engage in the job more and longer. Also, passion for a certain activity generally results in experiencing positive emotions during the activity. Such positive feelings can lead to positive experiences outside the workplace, and subsequently, increase life satisfaction (Vallerand, 2012; Curran et al., 2015). In addition, people who are passionate about their job do not use their personal sources to handle added levels of stress and anxiety, so they will still have sources and abilities at their disposal to participate in their personal life and enjoy it (Chummar et al., 2019). It has also been revealed that passion has a positive relationship with life satisfaction (Vallerand and Verner-Filion, 2013) and psychological well-being (Schellenberg and Bailis, 2015). In a meta-analysis, Pollack et al. (2020) report that passion at work is positively and significantly correlated with life satisfaction. However, no research has ever investigated the relationship between entrepreneurial passion and entrepreneurs’ satisfaction, which is surprising (Stephan, 2018). Based on the above discussion, the following hypothesis can be presented:

**H2:** Entrepreneurial passion is positively related to entrepreneurs’ well-being.

It has been established that job fit can positively affect people’s well-being because a positive perception of person-job fit increases the person’s satisfaction with her job. Also, when an individual perceives a closer fit between her abilities and job requests, she will experience less stress, fatigue, and concern and will do her job more effectively, which can, in turn, improve her well-being (Choi et al., 2017). This research assumes that this positive relationship between job fit and well-being is accounted for by entrepreneurial passion, at least partially. When entrepreneurs feel that their entrepreneurial job fits their ideal job, their entrepreneurial passion increases and their business forms a part of their identity. Consequently, they will enjoy their career and life to a greater extent.

**H3:** Entrepreneurial passion mediates the relationship between job fit and well-being.

### Well-Being and Entrepreneurial Persistence

Persistence is vital for entrepreneurial success. Launching, managing, and developing a business are faced with various challenges and barriers whose resolution requires putting a lot of energy, time, effort, and capital. Therefore, persistence throughout this process is a necessary dimension of entrepreneurship. Given the significance of persistence in entrepreneurship, it is crucial to recognize its antecedents (Cardon and Kirk, 2015). It is assumed in this research that well-being induced by job fit and high entrepreneurial passion acts as a key mechanism in accounting for persistent entrepreneurial activities. Previous research suggests that positive feelings, e.g., emotions, are important predictors of efforts for accomplishing goals (Custers and Aarts, 2005).

In this regard, Roland et al. (2016) state that subjective well-being affects persistence because positive feelings can promote the person’s intention and determination toward spending more effort and increase her expectations for success. Regarding entrepreneurship, these positive feelings can be influential on important dimensions of the entrepreneurial process, such as creativity, opportunity recognition, and the ability to respond to environmental changes (Baron, 2008). Also, good feelings throughout the entrepreneurial process can increase motivation, passion, and efforts toward entrepreneurial activities by controlling emotions and sudden incentives of the individual (Jia and Zhang, 2018). Marshall et al. (2020) found that well-being also had a similar effect on entrepreneurial persistence by enhancing it. Therefore, the following hypothesis is presented:

**H4:** Well-being is positively related to entrepreneurial persistence.

**FIGURE 1:**
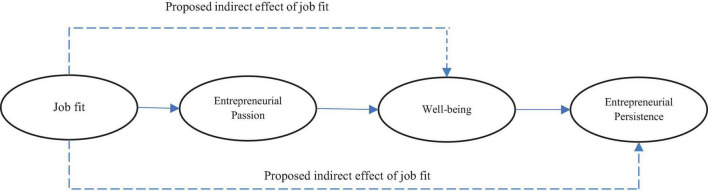
Conceptual framework.

Cardon and Kirk (2015) found a positive relationship between entrepreneurial passion and entrepreneurial persistence. To cope with challenges and problems in the entrepreneurial process, entrepreneurs should be passionate. They argue that both aspects of passion, i.e., identity and affective, can improve entrepreneurial persistence. When certain activities, such as entrepreneurial activities, are fitted with the individual’s identity and motivate positive emotions, entrepreneurial persistence increases. In this regard, Baron (2008) states that people usually attempt to preserve the positive state when faced with positive emotional situations, so they are more likely to be persistent in their entrepreneurial journey. The present research assumes that this important relationship between entrepreneurial passion and persistence is accounted for by positive emotions of well-being and delight, at least partially. When entrepreneurs are passionate about doing entrepreneurial activities, their well-being increases and they are more likely to spend more effort for success (Figure 1). Therefore, the following hypothesis is presented.

**H5:** Well-being mediates the relationship between entrepreneurial passion (as a result of job fit) and entrepreneurial persistence.

## Research Methodology

### Iranian Context

During the past decade in Iran, the government has developed a keen interest in the stimulation of entrepreneurship as an important mechanism for job creation, poverty reduction, and economic growth (Salamzadeh et al., 2017; Salamzadeh and Kesim, 2017; Karimi et al., 2021). According to the Statistics Center of Iran (2016), about 21 million people (26% of the total population) reside in the rural areas of Iran. A critical problem of Iran in recent decades has been the unemployment of rural people, which has encouraged their migration to urban areas so that a lot of villages have become desolated (Karimi et al., 2010). For this reason, the Iranian government has taken measures to alleviate poverty and increase employment and entrepreneurship in rural areas in order to develop rural areas. For example, the establishment of the Rural Women Microcredit Fund is one of the plans the government has implemented since 2000 to promote rural women’s empowerment and self-employment. In addition, it has recently allocated one and half a billion dollars from the National Development Fund for rural development in the country. It has also provided 4% interest loans to rural entrepreneurs as part of the Sixth National Development Plan (Soleymani et al., 2021). Despite the measures taken, promoting rural entrepreneurship and rural development in Iran needs more attention from policymakers, practitioners, and researchers. Based on the literature (Helliwell et al., 2017; Bhuiyan and Ivlevs, 2019), well-being is an important outcome that is closely related to poverty reduction and welfare in rural areas.

### Participants and Procedure

The research adopted quantitative methods to study rural entrepreneurs in Nahavand County in Hamedan Province, western Iran. It also employed cross-sectional data to investigate the relationships of the research model variables. A sample of 120 owners of small-sized rural businesses (with employee size of <50 people) was taken by the convenience sampling technique (due to the lack of a certain sampling framework) in the rural area of the county. Data were collected with a questionnaire. Since the scales used in the questionnaire were originally in English, they were translated into Persian using Brislin (1970) procedure. To emphasize the research importance, the research goal and the questionnaire content were explained to the participant entrepreneurs, and after their agreement, the data collection process was initiated. It took almost 1 month to finish. The participants voluntarily filled out a paper-and-pencil questionnaire anonymously at their workplace in May 2021. A cover letter appended to each questionnaire explained the research goal and how to fill the questionnaire. The participants were assured that the responses would be kept confidential.

A total of 120 questionnaires were distributed among the business owners, of which 110 questionnaires were returned. After the incomplete questionnaires were discarded, 102 questionnaires remained for statistical analysis. Research with even smaller sample sizes is commonplace in entrepreneurial research (e.g., Short et al., 2010; Cardon and Kirk, 2015). In addition, the PLS-SEM minimum sample size is estimated using the “10-times rule”, which was proposed by Barclay et al. (1995). This rule states that the minimum size should be ten times the largest number of paths directed to a single construct. “Work engagement” and “needs satisfaction” in the current study have the largest number of items (9); therefore, the minimum sample size should be 90 respondents.

In the final sample, the mean age of the respondents was 35.07 years and 68.6 percent of them were male. In terms of the educational level, 16.7 percent was under diploma, 38.2 percent had a diploma, and 45.1 percent had an academic degree. The average year of doing business at the time of the interviews was 5.66, and the average number of employees was 5.63. All studied businesses had less than 50 employees as per the definition of a small business in Iran. Among the businesses, 57.4 percent operated in the production sector, 37.3 percent in the service sector, and 4.9 percent in the financial/insurance sector.

### Measures

#### Job Fit

Following de Mol et al. (2018), job fit was measured using the General Job Fit Scale of Burnette and Pollack (2013). The respondents were asked to think about their current entrepreneurial job and express the degree of their agreement or disagreement with the relevant six items. An example item was: “My current job is as close to ideal as I ever expect to find.”

#### Entrepreneurial Passion

To measure entrepreneurial passion, following previous studies (Murnieks et al., 2014; Dalborg and Wincent, 2015), a seven-item scale for harmonious passion developed by Vallerand et al. (2003) was used. Sample items included: “For me, being an entrepreneur is a passion” and “I am completely taken with being an entrepreneur.” Respondents were asked to indicate their agreement with each item on a typical five-level Likert scale from totally disagree (1) to totally agree (5).

#### Well-Being

Following Shir et al. (2019), well-being was assessed using three global scales: (1) life satisfaction, (2) global happiness, and (3) subjective vitality. The variable life satisfaction was measured through the question “All things considered, how dissatisfied or satisfied you with your life as a whole are these days?” on a 5-point rating scale from 1 (not at all satisfied) to 5 (very satisfied). Global happiness was measured with the question: “All things considered; how happy would you say you are?” on a 5-point rating scale from 1 (not at all happy) to 5 (very happy). Finally, subjective vitality was measured using the item “I feel alive and vital” on a 5-point rating scale from 1 (not at all true) to 5 (very true).

#### Entrepreneurial Persistence

Entrepreneurial persistence was measured by using a six-item scale developed by Baum and Locke (2004). This scale has been used in many studies (Cardon and Kirk, 2015; Marshall et al., 2020). A sample item was: “I continue to work on hard projects even when others oppose me”.

### Control Variables

Based on the existing literature (e.g., Cardon and Kirk, 2015; Shir et al., 2019; Brieger et al., 2021), six control variables were used in this study. These were entrepreneurs’ age (year), gender (1 = female, 2 = male), education (1 = under high school diploma, 2 = high school diploma, 3 = university degree), marital status (1 = single, 2 = married), business age (year) and business size (number of employees).

### Data Analysis

Structural equation modeling (SEM) was used to analyze the data and test the research model for which the SmartPLS 3 software package was employed. This technique seemed more appropriate given the prospective nature of the research and its small sample size. At first, the validity and reliability of the research variables were tested, and then, the structural model was analyzed.

## Results

Table 1 presents the means, standard deviations, and correlations for the variables included in the research. Expectedly, all model variables had positive and significant correlations with one another. According to the relatively strong correlation between job fit, passion, well-being, and entrepreneurial persistence, multicollinearity was assessed (Hair et al., 2021). All variation inflation factor (VIF) values were <0.2 for these constructs, suggesting the lack of multicollinearity.

**TABLE 1:** Means, standard deviations, and correlations among variables.

Variables	Mean	S.D.	1	2	3	4	5	6	7	8	9
1. Age	35.7	9.36									
2. Gender	1.31	0.47	−*0*.35**								
3. Education	2.28	0.73	−*0*.*07*	−*0*.*18*							
4. Marital status	1.73	0.47	0.39**	0.04	0.09						
5. Business size	5.63	6.86	0.02	−*0*.*07*	−*0*.*11*	−*0*.*04*					
6. Business age	5.66	4.55	0.04	−*0*.*09*	0.06	0.09	0.05				
7. Job fit	3.17	0.82	0.09	−*0*.*01*	0.08	0.27**	−*0*.*03*	0.09			
8. Passion	3.86	0.74	0.11	−*0*.*05*	0.1	0.41**	−*0*.*09*	−*0*.*07*	0.59**		
9. Well-being	2.06	0.39	0.22	−*0*.*1*	0.04	0.23*	−*0*.20*	0.1	0.49**	0.51**	
10. Persistence	3.56	0.84	−*0*.*01*	0.01	−*0*.*04*	0.15	−*0*.*08*	0.11	0.39**	0.48**	0.41*

*p < 0.05; **p < 0.01.

### Assessment of Measurement Model

The reliability of the constructs was checked by Cronbach’s alpha and composite reliability (Hair et al., 2021). The results in Table 2 show that the coefficients of Cronbach’s alpha and composite reliability were higher than the acceptable level of 0.7 for all constructs. The convergent validity of the constructs was investigated by average variance extracted (AVE), which was found to be in the range of 0.52–0.64, showing that all values were higher than the acceptable level of 0.5. The HTMT approach was employed to study the divergent validity (Henseler et al., 2015). Based on the results, all HTMT values were lower than the acceptable level of 0.85, reflecting the divergent validity of the research scales (Kline, 2015).

**TABLE 2:** Assessment results of the measurement and structural models.

	Measurement model						Structural model	
					
Variable	α	CR	AVE	HTMT				
				1	2	3	*Q* ^2^	*R* ^2^
1. Job fit	0.87	0.9	0.58				–	–
2. Entrepreneurial passion	0.86	0.9	0.59	0.69			0.21	0.39
3. Entrepreneurial well-being	0.71	0.84	0.64	0.62	0.65		0.16	0.27
4. Entrepreneurial persistence	0.7	0.81	0.52	0.51	0.65	0.55	0.07	0.16

*α: Cronbach’s alpha; AVE: average variance extracted; CR: composite reliability; Q^2^: predictive relevance; R^2^: coefficient of determination.*

### Assessment of Structural Model

After the measurement model was confirmed to be valid and reliable, the structural model was examined. Before assessing this model, its fit was checked by SMRE assessment (Henseler et al., 2016). The PLS results showed that the SRMR value was 0.09, which is smaller than the threshold value of 0.10 (Hair et al., 2021). So, the overall fit of the structural model was confirmed. In the next step, the coefficients of determination (R^2^) were calculated for the endogenous constructs (passion, well-being, and entrepreneurial persistence). According to Cohen (1998), the R^2^ values of 0.60, 0.33, and 0.19 represent strong, moderate, and weak values in behavioral science research, respectively. The R^2^ value was 0.39 for entrepreneurial passion, implying its moderate predicting power. However, it was 0.27 and 0.19 for well-being and entrepreneurial persistence, respectively, showing their weak predicting level. Furthermore, the Stone-Geisser test indicated that the Q^2^ values were >0 for all latent variables, which supported the predictive relevance of the model (Table 2).

To assess the structural model, the bootstrapping method with 500 resamples was implemented (Hair et al., 2021). Table 3 presents the results of hypothesis testing including the effect sizes (*f*^2^). The *f*^2^ values of 0.35, 0.15, and 0.02 are assumed to be big, moderate, and small (Cohen, 1998). The results showed that job fit had a positive and significant relationship with entrepreneurial passion (β = 0.62, *p* < 0.01). The effect size was big for this relationship (*f^2^* = 0.63). So, H1 is supported. The results also showed the positive and significant relationship between entrepreneurial passion and entrepreneurial well-being (β = 0.52, *p* < 0.01), and its effect size was at a big level for this relationship (*f^2^* = 0.37). Finally, entrepreneurial well-being exhibited a positive and significant relationship with entrepreneurial persistence (β = 0.40, *p* < 0.01) with a moderate impact size (*f^2^* = 0.19). So, H4 is supported.

**TABLE 3:** Direct, indirect and total effects of the research model.

Hypotheses				β	*t values*	*CI*	*f* ^2^	Supported
	**Direct effects**							
H1	Job fit	→	Passion	0.62**	12.03	0.48–0.69	0.63	Yes
H2	Passion	→	Well-being	0.52**	5.4	0.31–0.70	0.37	Yes
H4	Well-being	→	Persistence	0.40**	4.43	19–0.56	0.19	Yes
	**Indirect effects**							
H3	Job fit → Passion	→	Well-being	0.32**	4.49	0.17–0.43		Yes
	Passion → Well-being	→	Persistence	0.21**	2.73	0.08–0.36		Yes
H5	Job fit → Passion →Well-being	→	Persistence	0.13**	2.56	0.05–0.23		

**p < 0.01.

To test the mediation hypotheses (H3 and H5), the procedure of mediation analysis was conducted in PLS using Nitzl et al.’s (2016) guideline. According to Nitzl et al. (2016), “testing the indirect effect a × b provides researchers with all information for testing mediation” (p. 1852); here “a” refers to the path between the independent variable and the mediator variable, while “b” represents the path between the mediator variable and the dependent variable. The significance test of the indirect effect showed that the indirect relationship between job fit and entrepreneurial well-being was significant due to the lack of zero in bias-corrected confidence intervals [β = 0.32, *CI* = (*0.17, 0.43*)]. Therefore, entrepreneurial passion mediates the relationship between job fit and entrepreneurial well-being, supporting H3. In addition, the results revealed the presence of a serial mediation model in which entrepreneurial passion and entrepreneurial well-being were important mechanisms that accounted for job fit and entrepreneurial persistence [β = 0.13, *CI* = (*0.05, 0.23*)]. So, H5 is confirmed too.

## Discussion and Conclusion

Although entrepreneurs’ well-being has been paid attention greatly in recent years, the antecedents and outcomes of well-being have been subject to limited studies. In this respect, Wiklund et al. (2019) motivate researchers to study mechanisms that lead to entrepreneurs’ well-being. They also state that little research has been conducted on well-being as a source or incentive for entrepreneurial action. In response to Wiklund et al.’s (2019) invitation and to fill the gap in the research literature, the present study investigated the antecedents (job fit and entrepreneurial passion) and outcomes (entrepreneurial persistence) of rural entrepreneurs’ well-being in Iran. As far as the authors know, this research is the first effort to study the antecedents and outcomes of rural entrepreneurs’ well-being in a developing country.

Based on the results, there is a positive relationship between job fit and entrepreneurial passion. In other words, entrepreneurs who feel that entrepreneurship fits their ideal job enjoy their business more so that the business forms a part of their identity. This is consistent with de Mol et al.’s (2018) study. We also found a positive relationship between entrepreneurial passion and entrepreneurs’ well-being. Entrepreneurs with more entrepreneurial passion are more satisfied with their job and life. According to the findings, well-being is also related to entrepreneurial persistence positively. This implies that the feeling of delight and satisfaction acts as an “incentive” for entrepreneurial persistence (Wiklund et al., 2019), which is in agreement with Marshall et al.’s (2018) study. Finally, the results showed a serial relationship between job fit and entrepreneurial persistence *via* entrepreneurial passion and well-being.

## Theoretical Implications

This research, which focused on studying and understanding rural entrepreneurs’ well-being in a developing country, contributes to both the entrepreneurship and well-being literature remarkably. Specifically, the research enriched the developing literature on the role of entrepreneurial passion. While entrepreneurship researchers have studied passion as an antecedent of startup performance and entrepreneurial persistence (Cardon et al., 2009; Ho and Pollack, 2014; Cardon and Kirk, 2015), few have addressed the relationship between entrepreneurial passion and well-being. The present study tried to answer Stephan’s (2018) call for research by exploring the direct relationship between passion on entrepreneurial well-being. It also tried to answer the call for further research by Wiklund et al. (2019) to predict entrepreneurial well-being. Furthermore, the present study helps the entrepreneurship literature by clarifying a relationship between entrepreneurial feeling (well-being) and entrepreneurial action (persistence). It was found that well-being can play the role of a ‘necessary psychological mechanism and resource (Wiklund et al., 2019, p. 584). In addition, this study focused exclusively on rural entrepreneurship that differs from urban entrepreneurship in context and features. Rural entrepreneurship scholars have suggested the development of theorizing on rural entrepreneurship (Müller and Korsgaard, 2018). This research is one of the first efforts to study how job fit and entrepreneurial passion act as the antecedents of well-being and how well-being, in turn, influences rural entrepreneurs’ persistence. It is crucial to study the antecedents and outcomes of rural entrepreneurs’ well-being both for researchers and for policymakers who intend to alleviate poverty and motivate socio-economic development in rural areas (Bhuiyan and Ivlevs, 2019). The present study, therefore, helps theorize on factors underpinning rural entrepreneurial outcomes.

## Practical Implications

From a practical perspective, the results of the present study have multiple implications. Given the role of job fit in improving entrepreneurial passion, well-being, and persistence, entrepreneurs should be adequately aware of entrepreneurial life and related activities from the very first moment for which they can consult with professional experts or take educational workshops. After initiating entrepreneurship, they should still put importance on job fit during business management and development and take tasks and responsibilities that are more fitted with their preferences, needs and professional nature. As has been stated by scholars, entrepreneurial passion is the heart of entrepreneurship (Cardon et al., 2009). Researchers argue that entrepreneurial passion is not merely a personality characteristic; rather, it is a unique emotional experience related to the entrepreneur’s personal nature and the type of role she is involved in Cardon et al. (2009). So, it lends itself to change and improvement, which can eventually result in the improvement of entrepreneurial well-being and persistence. A better understanding of the effects of entrepreneurial passion can help entrepreneurs to harness and leverage it to accomplish their goals.

Given the significance of well-being and its role in the positive outcomes of entrepreneurship, such as entrepreneurial persistence, entrepreneurs are suggested to put effort into preserving and improving their well-being, especially their life satisfaction, due to the role it plays in their job success. Entrepreneurs may not think about their life satisfaction separately during their business administration. Neglecting well-being can have serious consequences for the entrepreneur’s business. Research shows that life satisfaction is influenced by both personal factors in people’s lives and the social conditions and society (Diener et al., 2013). This means that a good life is not created in an isolated environment, but is shaped within social relations and interactions with others. Therefore, instead of merely focusing on their businesses, entrepreneurs should be able to invest in their social lives (Lindblom et al., 2020). The theoretical perspectives and empirical data also suggest that satisfaction and delight can beimproved in the long run by deliberate actions and exercises (Seligman, 2004; Lyubomirsky et al., 2005; Diener et al., 2006; Dijkhuizen et al., 2018). Entrepreneurs should therefore consider these activities and exercises for improving their well-being.

This study has also implications for policymakers who seek to encourage entrepreneurship in rural areas and understand what factors affect entrepreneurs’ well-being and persistence. Recent research suggests that policymakers can enhance both the number and well-being of start-ups leading them through strengthening the rule of law (Stephan et al., 2022). The results confirm the view that entrepreneurial passion will be an important parameter in the well-being and persistence configuration. The literature shows that entrepreneurial passion is trainable (e.g., Gielnik et al., 2017). Therefore, policymakers who take charge of rural entrepreneurship can tailor appropriate training programs for entrepreneurs to promote their entrepreneurial passion.

## Limitations and Future Research

As with other studies, this research is subject to some limitations. First, the research adopted a cross-sectional design, so it could not infer the causal relations of the variables. However, recent research has stated that cross-sectional designs have been criticized excessively (e.g., Spector, 2019). Nonetheless, future research is recommended to use longitudinal or experimental design to assess all antecedent and outcome variables to improve our understanding of these relationships. Second, this research used a self-reported questionnaire, so common method bias can be a source of concern. Although we followed recommended procedures to reduce this bias (Podsakoff et al., 2003), the findings should be treated with caution. Also, since psychological constructs such as entrepreneurial passion and life satisfaction are based on a subjective experience, self-reports are preferred as they provide more reliable data. Third, a convenience sample of rural entrepreneurs selected from Nahavand County was used. This is a common sampling technique in entrepreneurship research (Karimi et al., 2021; Vörös and Lukovszki, 2021; Wach et al., 2021) and can generate quality data despite its constraints. However, this limits the generalizability of the results. In addition, the study focused on rural entrepreneurs. So, caution should be exercised in generalizing its results to other fields. The distinctive traits of rural communities in Iran and their culture and lifestyle may account for the research findings. Further research is necessary to understand how the rural environment in Iran can affect entrepreneurial passion, well-being, and persistence. In addition, comparative studies should be conducted in other countries. Finally, well-being is important not only for people but also for society (Diener and Seligman, 2004) because it is accompanied by a wide range of positive outcomes such as entrepreneurial success and higher career performance (Dijkhuizen et al., 2018). Thus, this still needs to be scrutinized by researchers who can focus on the effect of other personal and environmental variables on it and its outcomes.

## Data Availability Statement

The raw data supporting the conclusions of this article will be made available by the authors, without undue reservation.

## Ethics Statement

Ethical review and approval was not required for the study on human participants in accordance with the local legislation and institutional requirements. Written informed consent for participation was not required for this study in accordance with the national legislation and the institutional requirements.

## Author Contributions

Both authors listed have made a substantial, direct, and intellectual contribution to the work, and approved it for publication.

## Conflict of Interest

The authors declare that the research was conducted in the absence of any commercial or financial relationships that could be construed as a potential conflict of interest.

## Publisher’s Note

All claims expressed in this article are solely those of the authors and do not necessarily represent those of their affiliated organizations, or those of the publisher, the editors and the reviewers. Any product that may be evaluated in this article, or claim that may be made by its manufacturer, is not guaranteed or endorsed by the publisher.
